# Supplementary material for: Synthetic G-quadruplex components for predictable, precise two-level control of mammalian recombinant protein expression
Source: Nucleic Acids Res. 2025 Jul 30;53(14):gkaf732. doi: 10.1093/nar/gkaf732 (PMC12309364; doi:10.1093/nar/gkaf732)
Supplement: gkaf732_Supplemental_File [file gkaf732_supplemental_file.pdf]

Supplementary Table S1. Synthetic G-quadruplex motif sequences. Components utilized in experiments to mechanistically dissect library function and validate performance in bioindustrial applications (i.e. Figs 3-5) are shown in bold. Sequences used as oligonucleotides in CD experiments were flanked by a single thymine at both ends.

**Motif number**	**Sequence**	**Component name**
DNA1	$G_2AG_2CG_2TG_2$	
DNA2	$G_3AG_3CG_3TG_3$	
DNA3	$G_4AG_4CG_4TG_4$	
DNA4	$G_5AG_5CG_5TG_5$	
DNA5	$G_6AG_6CG_6TG_6$	
DNA6	$G_2ACG_2CTG_2TAG_2$	
DNA7	$G_3ACG_3CTG_3TAG_3$	
**DNA8**	**$G_4ACG_4CTG_4TAG_4$**	**DNA.75REU**
DNA9	$G_5ACG_5CTG_5TAG_5$	
**DNA10**	**$G_6ACG_6CTG_6TAG_6$**	**DNA.45REU**
**DNA11**	**$G_2ACTG_2CTAG_2TACG_2$**	**DNA.95REU**
DNA12	$G_3ACTG_3CTAG_3TACG_3$	
**DNA13**	**$G_4ACTG_4CTAG_4TACG_4$**	**DNA.70REU**
DNA14	$G_5ACTG_5CTAG_5TACG_5$	
**DNA15**	**$G_6ACTG_6CTAG_6TACG_6$**	**DNA.50REU**
DNA16	$G_2ACTAG_2CTACG_2TACTG_2$	
DNA17	$G_3ACTAG_3CTACG_3TACTG_3$	
DNA18	$G_4ACTAG_4CTACG_4TACTG_4$	
DNA19	$G_5ACTAG_5CTACG_5TACTG_5$	
DNA20	$G_6ACTAG_6CTACG_6TACTG_6$	
DNA21	$G_2ACTACG_2CTACTG_2TACTAG_2$	
DNA22	$G_3ACTACG_3CTACTG_3TACTAG_3$	
DNA23	$G_4ACTACG_4CTACTG_4TACTAG_4$	
DNA24	$G_5ACTACG_5CTACTG_5TACTAG_5$	
**DNA25**	**$G_6ACTACG_6CTACTG_6TACTAG_6$**	**DNA.50REU.2**
DNA26	$G_2ACG_2CTG_2TAG_2ACG_2$	
DNA27	$G_3ACG_3CTG_3TAG_3ACG_3$	
DNA28	$G_4ACG_4CTG_4TAG_4ACG_4$	
DNA29	$G_5ACG_5CTG_5TAG_5ACG_5$	
**DNA30**	**$G_6ACG_6CTG_6TAG_6ACG_6$**	**DNA.30REU**
DNA31	$G_2ACG_2CTG_2TAG_2ACG_2CTG_2$	
DNA32	$G_3ACG_3CTG_3TAG_3ACG_3CTG_3$	
DNA33	$G_4ACG_4CTG_4TAG_4ACG_4CTG_4$	
DNA34	$G_5ACG_5CTG_5TAG_5ACG_5CTG_5$	
**DNA35**	**$G_6ACG_6CTG_6TAG_6ACG_6CTG_6$**	**DNA.20REU**
DNA36	$G_2ACG_2CTG_2TAG_2ACG_2CTG_2TAG_2$	
DNA37	$G_3ACG_3CTG_3TAG_3ACG_3CTG_3TAG_3$	
DNA38	$G_4ACG_4CTG_4TAG_4ACG_4CTG_4TAG_4$	
DNA39	$G_5ACG_5CTG_5TAG_5ACG_5CTG_5TAG_5$	
**DNA40**	**$G_6ACG_6CTG_6TAG_6ACG_6CTG_6TAG_6$**	**DNA.10REU**
DNA41	$G_3AAAG_3AAAG_3AAAG_3$	
DNA42	$G_3AACG_3AACG_3AACG$	
DNA43	$G_3AATG_3AATG_3AATG$	
DNA44	$G_3ACAG_3ACAG_3ACAG$	
DNA45	$G_3ACCG_3ACCG_3ACCG$	
DNA46	$G_3ACTG_3ACTG_3ACTG$	
**DNA47**	**$G_3ATAG_3ATAG_3ATAG$**	**DNA.70REU.2**
DNA48	$G_3ATCG_3ATCG_3ATCG$	
DNA49	$G_3ATTG_3ATTG_3ATTG$	
DNA50	$G_3CAAG_3CAAG_3CAAG$	
DNA51	$G_3CACG_3CACG_3CACG$	
DNA52	$G_3CATG_3CATG_3CATG$	

DNA53	$G_3CCAG_3CCAG_3CCAG$	
DNA54	$G_3CCCG_3CCCG_3CCCG$	
DNA55	$G_3CCTG_3CCTG_3CCTG$	
DNA56	$G_3CTAG_3CTAG_3CTAG$	
DNA57	$G_3CTCG_3CTCG_3CTCG$	
DNA58	$G_3CTTG_3CTTG_3CTTG$	
DNA59	$G_3TAAG_3TAAG_3TAAG$	
DNA60	$G_3TACG_3TACG_3TACG$	
DNA61	$G_3TATG_3TATG_3TATG$	
DNA62	$G_3TCAG_3TCAG_3TCAG$	
**DNA63**	**$G_3TCCG_3TCCG_3TCCG$**	**DNA.70REU.3**
DNA64	$G_3TCTG_3TCTG_3TCTG$	
**DNA65**	**$G_3TTAG_3TTAG_3TTAG$**	**DNA.70REU.4**
DNA66	$G_3TTCG_3TTCG_3TTCG$	
DNA67	$G_3TTTG_3TTTG_3TTTG$	
DNAmut1	AGTACTAGTCTAAGTTACAGT	
DNAmut2	AAGTTACTAAGTTCTAAAGTTTACAAGTT	
**RNA1**	**$G_2AG_2CG_2UG_2$**	**RNA.90REU**
**RNA2**	**$G_3AG_3CG_3UG_3$**	**RNA.70REU**
RNA3	$G_4AG_4CG_4UG_4$	
**RNA4**	**$G_5AG_5CG_5UG_5$**	**RNA.25REU**
RNA5	$G_6AG_6CG_6UG_6$	
RNA6	$G_2ACG_2CUG_2UAG_2$	
**RNA7**	**$G_3ACG_3CUG_3UAG_3$**	**RNA.80REU**
RNA8	$G_4ACG_4CUG_4UAG_4$	
**RNA9**	**$G_5ACG_5CUG_5UAG_5$**	**RNA.50REU**
RNA10	$G_6ACG_6CUG_6UAG_6$	
**RNA11**	**$G_2ACUG_2CUAG_2UACG_2$**	**RNA.90REU**
RNA12	$G_3ACUG_3CUAG_3UACG_3$	
**RNA13**	**$G_4ACUG_4CUAG_4UACG_4$**	**RNA.65REU**
RNA14	$G_5ACUG_5CUAG_5UACG_5$	
**RNA15**	**$G_6ACUG_6CUAG_6UACG_6$**	**RNA.40REU**
RNA16	$G_2ACUAG_2CUACG_2UACUG_2$	
RNA17	$G_3ACUAG_3CUACG_3UACUG_3$	
RNA18	$G_4ACUAG_4CUACG_4UACUG_4$	
RNA19	$G_5ACUAG_5CUACG_5UACUG_5$	
**RNA20**	**$G_6ACUAG_6CUACG_6UACUG_6$**	**RNA.35REU**
RNA21	$G_2ACUACG_2CUACUG_2UACUAG_2$	
RNA22	$G_3ACUACG_3CUACUG_3UACUAG_3$	
RNA23	$G_4ACUACG_4CUACUG_4UACUAG_4$	
RNA24	$G_5ACUACG_5CUACUG_5UACUAG_5$	
RNA25	$G_6ACUACG_6CUACUG_6UACUAG_6$	
RNA26	$G_2ACG_2CUG_2UAG_2ACG_2$	
RNA27	$G_3ACG_3CUG_3UAG_3ACG_3$	
RNA28	$G_4ACG_4CUG_4UAG_4ACG_4$	
RNA29	$G_5ACG_5CUG_5UAG_5ACG_5$	
**RNA30**	**$G_6ACG_6CUG_6UAG_6ACG_6$**	**RNA.15REU**
RNA31	$G_2ACG_2CUG_2UAG_2ACG_2CUG_2$	
RNA32	$G_3ACG_3CUG_3UAG_3ACG_3CUG_3$	
RNA33	$G_4ACG_4CUG_4UAG_4ACG_4CUG_4$	
RNA34	$G_5ACG_5CUG_5UAG_5ACG_5CUG_5$	
RNA35	$G_6ACG_6CUG_6UAG_6ACG_6CUG_6$	
RNA36	$G_2ACG_2CUG_2UAG_2ACG_2CUG_2UAG_2$	
**RNA37**	**$G_3ACG_3CUG_3UAG_3ACG_3CUG_3UAG_3$**	**RNA.50REU**
RNA38	$G_4ACG_4CUG_4UAG_4ACG_4CUG_4UAG_4$	
RNA39	$G_5ACG_5CUG_5UAG_5ACG_5CUG_5UAG_5$	
**RNA40**	**$G_6ACG_6CUG_6UAG_6ACG_6CUG_6UAG_6$**	**RNA.5REU**
RNA41	$G_3AAAG_3AAAG_3AAAG_3$	

RNA42	$G_3AACG_3AACG_3AACG$	
RNA43	$G_3AAUG_3AAUG_3AAUG$	
RNA44	$G_3ACAG_3ACAG_3ACAG$	
RNA45	$G_3ACCG_3ACCG_3ACCG$	
RNA46	$G_3ACUG_3ACUG_3ACUG$	
RNA47	$G_3AUAG_3AUAG_3AUAG$	
RNA48	$G_3AUCG_3AUCG_3AUCG$	
RNA49	$G_3AUUG_3AUUG_3AUUG$	
RNA50	$G_3CAAG_3CAAG_3CAAG$	
RNA51	$G_3CACG_3CACG_3CACG$	
RNA52	$G_3CAUG_3CAUG_3CAUG$	
RNA53	$G_3CCAG_3CCAG_3CCAG$	
RNA54	$G_3CCCG_3CCCG_3CCCG$	
RNA55	$G_3CCUG_3CCUG_3CCUG$	
RNA56	$G_3CUAG_3CUAG_3CUAG$	
RNA57	$G_3CUCG_3CUCG_3CUCG$	
RNA58	$G_3CUUG_3CUUG_3CUUG$	
RNA59	$G_3UAAG_3UAAG_3UAAG$	
RNA60	$G_3UACG_3UACG_3UACG$	
RNA61	$G_3UAUG_3UAUG_3UAUG$	
RNA62	$G_3UCAG_3UCAG_3UCAG$	
RNA63	$G_3UCCG_3UCCG_3UCCG$	
RNA64	$G_3UCUG_3UCUG_3UCUG$	
RNA65	$G_3UUAG_3UUAG_3UUAG$	
RNA66	$G_3UUCG_3UUCG_3UUCG$	
RNA67	$G_3UUUG_3UUUG_3UUUG$	
RNAmut1	AGUACUAGUCUAAGUUACAGU	
RNAmut2	AAGUUACUAAGUUCUAAAGUUUACAAGUU	

Supplementary Table S2. Sequences of endogenous G4-quadruplex motifs, and their mutated versions (G-to-A mutations underlined). Propensity of sequences to form G4 structures was predicted using QGRS mapper (49)

**Motif**	**Sequence**	**G-score**
c-myc wild type	TGGGGAGGGTGGGGAGGGTGGGGAAGG	41
c-myc mut1	TGAGGAGAGTGAGGAGAGTGAGGAAGG	16
c-myc mut2	TGAAGAGAATGAAGAGAATGAAGAAGG	0
Nras wild type	GGGAGGGGCGGGUCUGGG	40
Nras mut1	AAAAGGGGCGGGUCUGGG	18
Nras mut2	AAAAGAGGCGAGUCUGAG	0

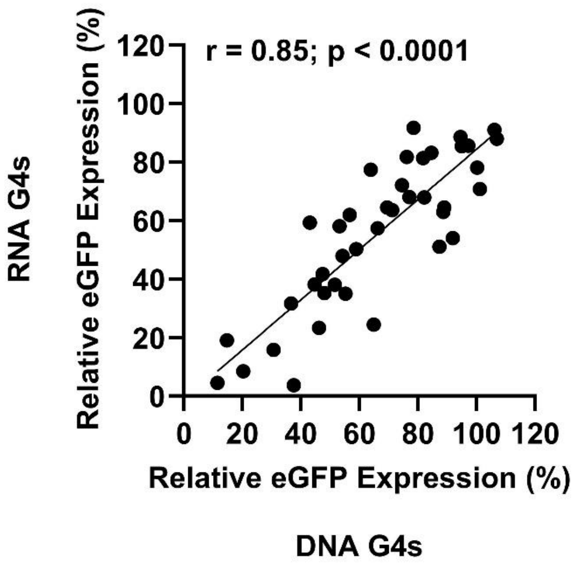

Supplementary Figure S1. Correlation between the inhibitory activity of synthetic G4 sequences when deployed at the DNA and RNA level. Synthetic G4 components (listed in Supplementary Table 1) with varying sequence feature compositions were inserted into positions -1 (DNA) and +6 (RNA) respectively in the BPCU (see Fig. 1) downstream of a CMV-IE1 proximal promoter in eGFP-reporter vectors. CHO cells were transfected with eGFP-reporter plasmids prior to protein quantification after 48 h. Data are expressed as a percentage of the production exhibited by the control CMV-IE1 proximal promoter-BPCU (ΔG4) construct. Values represent the mean of three independent experiments (n = 3, each performed in triplicate).

DNA

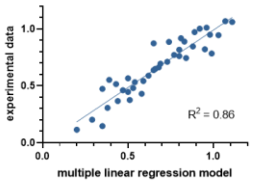


```
Residuals:
     Min        1Q    Median        3Q       Max
-0.193073 -0.059262 -0.005414  0.062684  0.235242

Coefficients:
            Estimate Std. Error t value Pr(>|t|)
(Intercept)  1.80875    0.10259  17.631  < 2e-16 ***
looplen     -0.04171    0.01363  -3.060  0.00417 **
Glen        -0.15117    0.01099 -13.758 6.62e-16 ***
Gnum        -0.09051    0.01524  -5.938 8.40e-07 ***
---
Signif. codes:  0 '***' 0.001 '**' 0.01 '*' 0.05 '.' 0.1 ' ' 1

Residual standard error: 0.09828 on 36 degrees of freedom
Multiple R-squared:  0.8624,	Adjusted R-squared:  0.8509
F-statistic:  75.2 on 3 and 36 DF,  p-value: 1.427e-15
```

RNA

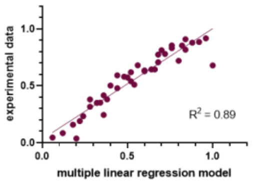


```
Residuals:
    Min       1Q   Median       3Q      Max
-0.32057 -0.04053  0.00984  0.05862  0.13283

Coefficients:
             Estimate Std. Error t value Pr(>|t|)
(Intercept)  1.365705   0.092645  14.741  < 2e-16 ***
looplen      0.037023   0.012311   3.007 0.004784 **
Glen        -0.155249   0.009923 -15.646  < 2e-16 ***
Gnum        -0.060118   0.013764  -4.368 0.000102 ***
---
Signif. codes:  0 '***' 0.001 '**' 0.01 '*' 0.05 '.' 0.1 ' ' 1

Residual standard error: 0.08875 on 36 degrees of freedom
Multiple R-squared:  0.8887,	Adjusted R-squared:  0.8794
F-statistic: 95.84 on 3 and 36 DF,  p-value: < 2.2e-16
```

$$y = 1.81 + (-0.04 \cdot x_1) + (-0.15 \cdot x_2) + (-0.09 \cdot x_3) \qquad y = 1.36 + (0.04 \cdot x_1) + (-0.16 \cdot x_2) + (-0.06 \cdot x_3)$$

x_1= loop length x_2= G-tract length x_3= G-tract number

Supplementary Figure S2. **Multiple linear regression models explaining synthetic G4 inhibitory activities as a function of their input design parameters**. Synthetic G4 components (listed in Supplementary Table 1) with varying sequence feature compositions were inserted into positions -1 (DNA) and +6 (RNA) respectively in the BPCU (see Fig. 1) downstream of a CMV-IE1 proximal promoter in eGFP-reporter vectors. CHO cells were transfected with eGFP-reporter plasmids prior to protein quantification after 48 h. Data were expressed as a percentage of the production exhibited by the control CMV-IE1 proximal promoter-BPCU (ΔG4) construct. Linear regression models were fit using relative expression units as the response variable and loop length, G-tract length and G-tract number as the predictor variables. Model summary data is shown, alongside plots of observed vs predicted values.

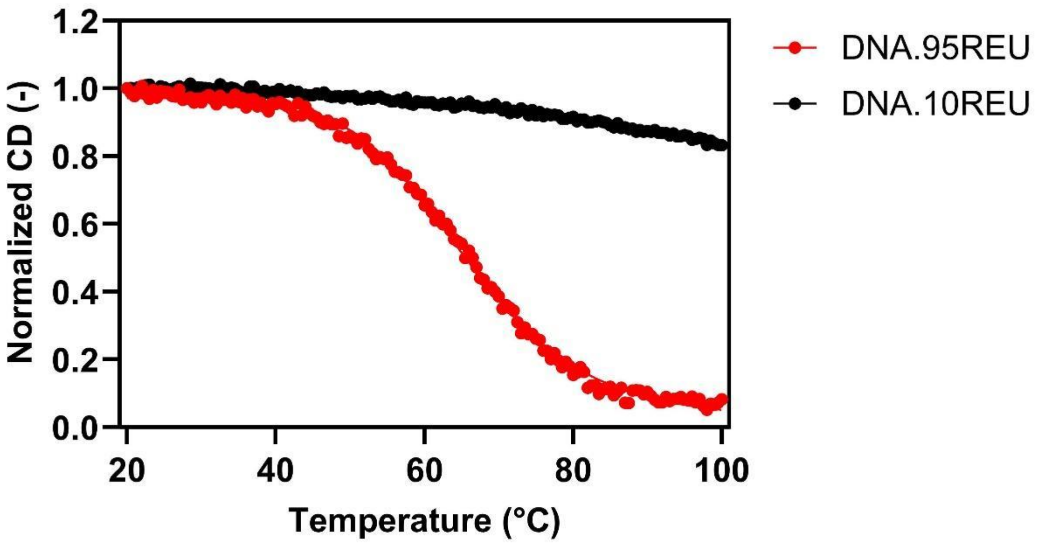

Supplementary Figure S3. Melting curve analysis of synthetic G4 DNA elements with high (DNA.10REU) and low (DNA.95REU) inhibitory activities. Analysis performed in 50 mM Tris-HCl buffer (pH 7.5) with 100 mM KCl.

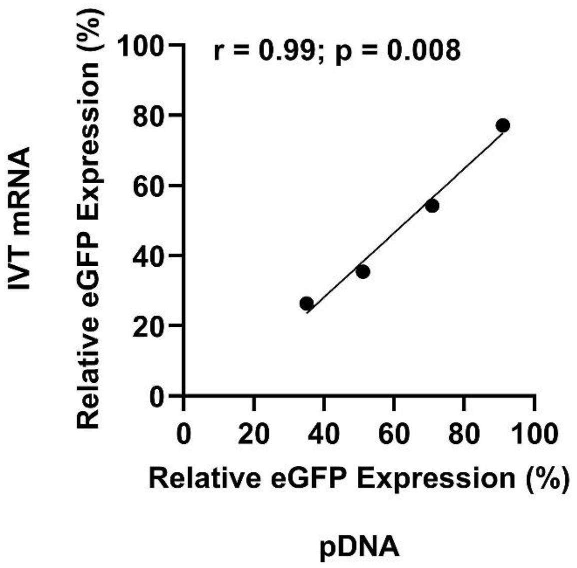

Supplementary Figure S4. Correlation between synthetic G4 component performance in DNA vector and mRNA transcript contexts. CHO cells were transfected with eGFP encoding reporter plasmids and mRNA molecules containing varying RNA G4 elements. eGFP levels were quantified 48 h post-transfection. Data are expressed as a percentage of the production exhibited by control BPCU DNA and RNA constructs (ΔG4). Values represent the mean of three independent experiments (n = 3, each performed in triplicate).

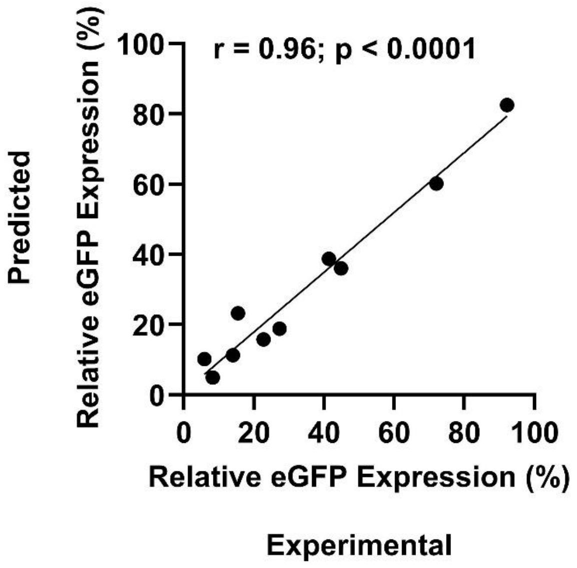

Supplementary Figure S5. Correlation between predicted and observed values when synthetic G4s were used in combination. CHO cells were transiently transfected with eGFP reporter plasmids containing varying combinations of DNA and RNA G4 elements. eGFP levels were quantified 48 h post-transfection and compared to those driven by predicted values, assuming constituent G4 pairs act synergistically. Values represent the mean of three independent experiments (n = 3, each performed in triplicate).

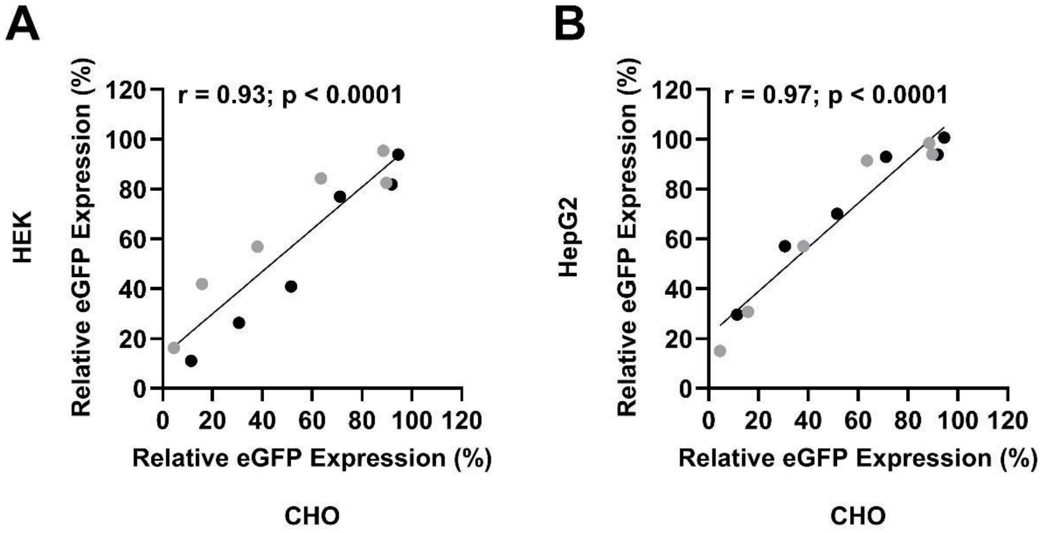

Supplementary Figure S6. Correlation between synthetic G4 regulatory activities in different cell hosts. eGFP-reporter plasmids containing DNA (black dots) or RNA (grey dots) G4 components with varying activities were transfected into CHO, HEK and HepG2 cells, prior to protein quantification 48 h later. Data are expressed as a percentage of the production exhibited by the control CMV-IE1 proximal promoter-BPCU (ΔG4) construct. Values represent the mean of three independent experiments (n = 3, each performed in triplicate).

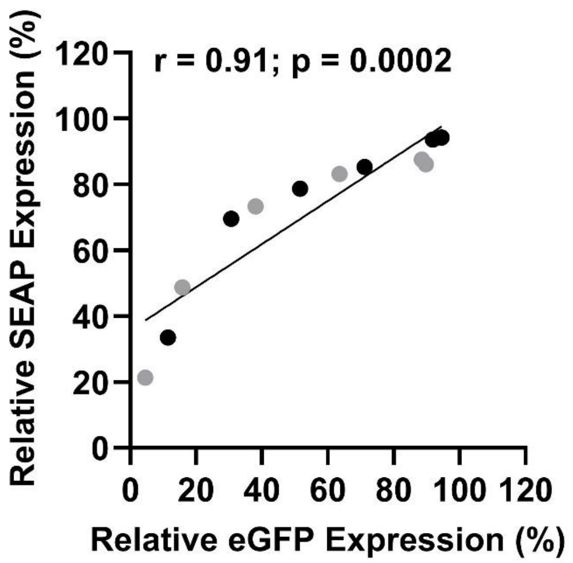

Supplementary Figure S7. Correlation between synthetic G4 regulatory activities in different recombinant protein contexts. CHO cells were transfected with reporter plasmids encoding eGFP or SEAP production under the control of varying-strength DNA (black dots) or RNA (grey dots) G4 elements. Data are expressed as a percentage of the production exhibited by the control CMV-IE1 proximal promoter-BPCU (ΔG4) construct. Values represent the mean of three independent experiments (n = 3, each performed in triplicate).
